# Early Comprehensive Kidney Care in Dialysis-Requiring Acute Kidney Injury Survivors: A Populational Study

**DOI:** 10.3389/fmed.2022.847462

**Published:** 2022-04-22

**Authors:** Chun-Yi Wu, Jia-Sin Liu, Cheng-Hsu Chen, Chun-Te Huang, Tung-Min Yu, Ya-Wen Chuang, Shih-Ting Huang, Chih-Cheng Hsu, Ming-Ju Wu

**Affiliations:** ^1^Division of Nephrology, Department of Internal Medicine, Taichung Veterans General Hospital, Taichung, Taiwan; ^2^Department of Nursing, Asia University, Taichung, Taiwan; ^3^Institute of Population Health Sciences, National Health Research Institutes, Miaoli, Taiwan; ^4^School of Medicine, College of Medicine, China Medical University, Taichung, Taiwan; ^5^Department of Post-baccalaureate Medicine, College of Medicine, National Chung Hsing University, Taichung, Taiwan; ^6^Department of Life Science, Tunghai University, Taichung, Taiwan; ^7^Department of Internal Medicine and Critical Care Medicine, Nephrology and Critical Care Medicine, Taichung Veterans General Hospital, Taichung, Taiwan; ^8^Graduate Institute of Biomedical Sciences, College of Medicine, China Medical University, Taichung, Taiwan; ^9^Department of Health Services Administration, China Medical University, Taichung, Taiwan; ^10^Department of Family Medicine, Min-Sheng General Hospital, Taoyuan, Taiwan; ^11^National Center for Geriatrics and Welfare Research, National Health Research Institutes, Miaoli, Taiwan; ^12^RongHsing Research Center for Translational Medicine, College of Life Sciences, National Chung Hsing University, Taichung, Taiwan; ^13^Ph.D. Program in Translational Medicine, National Chung Hsing University, Taichung, Taiwan; ^14^School of Medicine, Chung Shan Medical University, Taichung, Taiwan; ^15^Graduate Institute of Clinical Medical Sciences, College of Medicine, China Medical University, Taichung, Taiwan

**Keywords:** acute kidney injury, AKI follow-up, dialysis-requiring acute kidney injury, comprehensive kidney care, end-stage renal disease

## Abstract

**Background:**

For patients with Acute Kidney Injury (AKI), a strong and graded relationship exists between AKI severity and mortality. One of the most severe entities of AKI is Dialysis-Requiring Acute Kidney Injury (D-AKI), which is associated with high rates of mortality and end-stage renal disease (ESRD). For this high-risk population group, there is a lack of evidence regarding optimal post-AKI care. We propose that post-AKI care through the combined efforts of the nephrologist and the multidisciplinary care team may improve outcomes. Our aim here is to study for survivors of dialysis-requiring acute kidney injury, the effects of implementing early comprehensive kidney care.

**Methods:**

This is a retrospective longitudinal cohort study of Taiwanese through analyzing the National Health Insurance Research Database (NHIRD). We included patients with acute dialysis during hospitalization from January 1, 2015 to December 31, 2018. Propensity match was done at 1:1, including estimated glomerular filtration rate (eGFR) based on CKD-EPI which was performed due to large initial disparities between these two cohorts.

**Results:**

After the propensity match, each cohort had 4,988 patients. The mean eGFR based on CKD-EPI was 27.5 ml/min/1.73 m^2^, and the mean follow-up period was 1.4 years.

The hazard ratio for chronic dialysis or ESRD was 0.55 (95% CI, 0.49–0.62; *p* < 0.001). The hazard ratio for all-cause mortality was 0.79 (95% CI, 0.57–0.88; *p* < 0.001). Both outcomes favored early comprehensive kidney care.

**Conclusions:**

For survivors of dialysis-requiring acute kidney injury, early comprehensive kidney care significantly lowered risks of chronic dialysis and all-cause mortality.

## Introduction

Acute Kideny Injury (AKI) is a syndrome characterized by a rapid rise in serum creatinine level, a drop in urine output, or both. AKI is a common disorder with a high incidence rate of 18.3% from all hospitalizations (1). The incidence in the intensive care setting is even higher, at a staggering rate of 57.3% as reported in a multinational study (2).

A strong and graded relationship exists between AKI severity and mortality (3). Long-term complications of AKI include poor survival, progression to chronic kidney disease (CKD), and even deterioration to end-stage renal disease (ESRD) (4, 5). AKI individuals bear a 9-times higher risk of CKD, 3-times higher risk of ESRD, and a 2-times higher risk of premature death compared with matched patients without AKI (5).

Dialysis-Requiring Acute Kidney Injury (D-AKI) is one of the most severe entities of AKI KDIGO stage 3 (6), with high rates of mortality and ESRD (7, 8). A retrospective study found that 66% of D-AKI patients had no recovery in kidney function, and they required chronic dialysis. Whereas, only 19% of them recovered kidney function to the extent without the need of further dialysis. Furthermore, 16% of D-AKI patients died (7). In a study involving intensive care patients surviving D-AKI, the subsequent risk of ESRD for D-AKI patients was 8.5%, within 180 days following ICU admission, compared with 0.1% for non-D-AKI ICU patients. This difference corresponds to an adjusted HR of 105.6. Among patients who had survived 180 days after ICU admission without developing ESRD, the 181-day to 5-year ESRD risk was 3.8% for patients with D-AKI, compared with 0.3% for non-D-AKI ICU patients. The adjusted HR of 6.2 (8). In another single center study of 63 non-critically ill patients, the renal outcome was still severe but not as dim as the critically ill patients. Six patients (10%) presented early full renal recovery, and dialysis treatment was discontinued in 38 patients (60%); 25 patients (40%) required maintenance dialysis (9).

Regarding community follow-up after AKI hospitalization, there are currently no evidence guiding optimal care. Although nephrologists expressed that nephrology care was important for patients after hospitalization with AKI. Only a minority of patients actually received follow-up with a nephrologist (10). To make matters worse, a cross-sectional survey revealed that 80% of patients were unaware that they had ever experienced AKI (11). Many patients did not receive adequate post-AKI care despite their high risks of death way after apparent recovery from the initial insult. Thus, post-discharge AKI care represents an opportunity for the medical community to improve patient outcomes. Since only 8.5–25% of AKI survivors visit nephrologists (12, 13), and observational studies suggest an association between early nephrology follow-up and better clinical outcomes (12, 14, 15). In a similar study, dialysis-requiring AKI patients who had recovered without dialysis, only 40% visited a nephrologist within 90 days of hospital discharge. In this cohort, early nephrology follow-up after hospitalization was associated with a 24% reduction in mortality (14). A randomized controlled trial, the Nephrologist Follow-up vs. Usual Care after an Acute Kidney Injury Hospitalization (FUSION) attempted to study this issue. However, due to feasibility barriers, 10 of the 34 patients of the intervention group that was assigned to the nephrology follow-up did not complete their follow-up. Hence, the trial failed to draw any conclusions (16).

AKI and its subsequent complications: CKD and ESRD bear tremendous economic burden to the health care system (17, 18). ESRD patients constitute 0.4% of the total population in Taiwan, but account for 8.7–9.3% of total health expenditure of the national health insurance system.

The Taiwan nationwide pre-end-stage renal disease (pre-ESRD) pay-for-performance program (P4P) was implemented since 2006, with the aim to improve quality of CKD care in patients with CKD stages 3B, 4, and 5 (19–21). Centered on the nephrologist, the pre-ESRD program also combines the effort of a multidisciplinary care team consisting of renal nurses, renal pharmacists, and dietitians to provide kidney care. Enrollment to the program is initiated exclusively by the nephrologist. Follow-up and education by the nephrologist and multidisciplinary care team on a regular 3 months basis is mandated for case payment. Previous studies show that enrollment into the pre-ESRD program can lower the incidence of dialysis and reduce mortality (22). Participants in the pre-ESRD program reduced 68.4% of their 4-year healthcare expenditure, and subsequently 22% reduction in 3-year mortality after dialysis, when compared with non-participants (20, 21).

Our hypothesis is that early intervention via the combined efforts of the nephrologist and the multidisciplinary care team improves renal outcomes and mortality of dialysis-free D-AKI patients.

The aim of this study is to determine effects of early comprehensive kidney care for survivors of dialysis-requiring acute kidney injury.

## Patients and Methods

### Data Sources

We used Taiwan's National Health Insurance Research Database (NHIRD) as a source of data with the approval of the Research Ethics Committee of the National Health Research Institutes (EC1060704-E). The National Health Insurance (NHI) program in Taiwan includes more than 255 hospitals and about 400 dialysis clinics that provide care service of dialysis (23, 24). Despite the name of national health “insurance,” the program is in practice obligatory, encompassing 99% of the Taiwanese population. For research purposes, the National Health Insurance administration established the NHIRD, which contains information regarding disease diagnoses, laboratory tests, drug prescriptions, operation codes, medical procedures, and reimbursement costs for all outpatient visits and hospitalizations. Disease diagnoses are assigned according to the disease diagnoses and procedures are coded according to the International Classification of Diseases, 9th Revision, Clinical Modification (ICD-9-CM) before 2015, and after 2015 according to the International Classification of Diseases, 10th Revision, Clinical Modification (ICD-10-CM). NHIRD database, since 2015, also contains some specific biochemical values in clinical care, such as blood tests, and urine analysis data. The personal information in the NHIRD and linked database are encrypted to protect privacy.

### Study Design and Participants

This was a retrospective longitudinal cohort study to determine effects of combining the nephrologist-centered kidney care and the multidisciplinary care team on survivors of dialysis-requiring acute kidney injury. We included patients with acute dialysis during hospitalization from January 1, 2015 to December 31, 2018. These patients also fulfilled a second criteria of not receiving chronic dialysis or death within 90 days of discharge. The date of 90 days after D-AKI discharge is defined as the index date. Subjects who had enrolled in the pre-ESRD program before the index date were enrolled into the comprehensive kidney care group, and others into the standard care group. Dialysis procedures and the pre-ESRD program were reimbursed via the NHI, which validated a high accuracy of procedure execution via routine data audits submitted by healthcare institutions and providers.

Subjects were excluded if their birthdate or gender records were missing, or if the age was >100 or <20 years old at D-AKI, or without serum creatinine value within 90 days after discharge.

The Taiwan pre-ESRD program provides kidney care centered on the nephrologist and the multidisciplinary care team consisting of renal nurses, renal pharmacists, and dietitians (19, 25). All non-dialysis patients with two serial serum creatinine examinations that reveal eGFR <45 may be eligible for enrollment into the program. However, the enrollment process must be initiated by the nephrologist, which may require referral to a nephrologist if the patient receives follow-up at other specialties. After enrollment, blood tests and follow-up by the nephrologist and multidisciplinary care team on a regular 3 months interval is mandated. Due to the fact that the pre-ESRD program is a paid for performance program, all requirements must be met for case payment. Thus, the pre-ESRD program is a suitable marker of comprehensive kidney care.

### Baseline Characteristics

The comorbidity of patients was based on their inpatient and outpatient records 1 year prior to the initial dialysis. Comorbidities included the following: diabetes mellitus, hypertension, dyslipidemia, cerebrovascular accident (CVA), congestive heart failure (CHF), cardiovascular disease (CVD), hepatitis B virus (HBV), hepatitis C virus (HCV), chronic obstructive pulmonary disease (COPD), acute kidney disease (AKI), and cancer. Comorbidities were identified if the diagnosis occurred twice in patient's outpatient records, or once in hospitalization records within 1 year prior to the index date. The NHI administration routinely audits data submitted by healthcare institutions and providers to detect possible frauds. In addition, the NHI is the sole insurance carrier of the covered healthcare in Taiwan. To avoid the rejection of reimbursements from the NHI, physicians follow stringent clinical guidelines suggested by the administration. The accuracy of diagnoses of has been validated in previous studies (26, 27). The Charlson comorbidity index scores were calculated (28). The serum creatinine level selected was based on the latest serum creatinine examination obtained within 90 days after discharge. The subsequent estimated Glomerular filtration rate (eGFR) was according to the CKD-EPI formula (29). We also stratified patients into various CKD stages (3A, 3B, 4, and 5) based on eGFR values.

### Outcome Variables: Chronic Dialysis Rate and All-Cause Mortality

The observation period started from the index date until December 31, 2018. Chronic dialysis was defined as continuous treatment with dialysis uninterruptedly for >3 months. The all-cause mortality was the pronouncement of death in the hospital, or the date NHI provided in death records of subjects after hospitalization.

### Statistical Analyses

The chi-square test was applied to compare the characteristics of comprehensive kidney care and standard care subjects. The independent *t*-test was used to compare the age and renal function of comprehensive kidney care and standard care subjects. All characteristics were compared by the standard mean difference. In the multivariate Cox proportional hazards regression model, effects of comprehensive kidney care were adjusted for age, sex, comorbidity, and renal function. Results were expressed as hazard ratio (HR) as compared among subjects enrolled in comprehensive kidney care. There was no violation of the assumption that the constant hazard ratio (HR) over time according to estimated log-log survival curves among all time-independent covariates. Kaplan-Meier and log-rank tests were used to assess differences between cumulative chronic dialysis rate and all-cause mortality. Adjusted HR and 95% confidence interval (CI) for chronic dialysis and all-cause mortality of the comprehensive kidney care patients were analyzed based on participants' characteristics. After adjusting covariates, the cumulative hazard of chronic dialysis and all-cause mortality were compared between the enrolled comprehensive kidney care and standard care by using the Cox proportional-hazard regression model. We adopted a 1:1 propensity score-matched analysis. We thus calculated a propensity score for the likelihood of using enrolled into comprehensive kidney care by logistic regression analysis, conditioned on the baseline covariates demographic characteristics, prevalence comorbidity, renal function, and CKD stage. In all-cause mortality risk analysis, the dialysis vintage was considered as the time-dependent covariate to calculate adjusted hazard ratio (HR). Further, we also used a robust sandwich covariance matrix estimate to account for the intracluster dependence on AKI admission of hospitals or clinics. The subgroup analysis separately compared chronic dialysis rate and all-cause mortality risk according to age, gender, comorbidity, and CKD stage. Statistical significance was set at *p* < 0.05, two-tailed test. Data compilation and statistical analyses were done with STATA 16.1 (STATA Corp, College Station, TX) and SAS 9.4 (SAS Institute Inc., Cary, NC).

## Results

**Figure 1:**
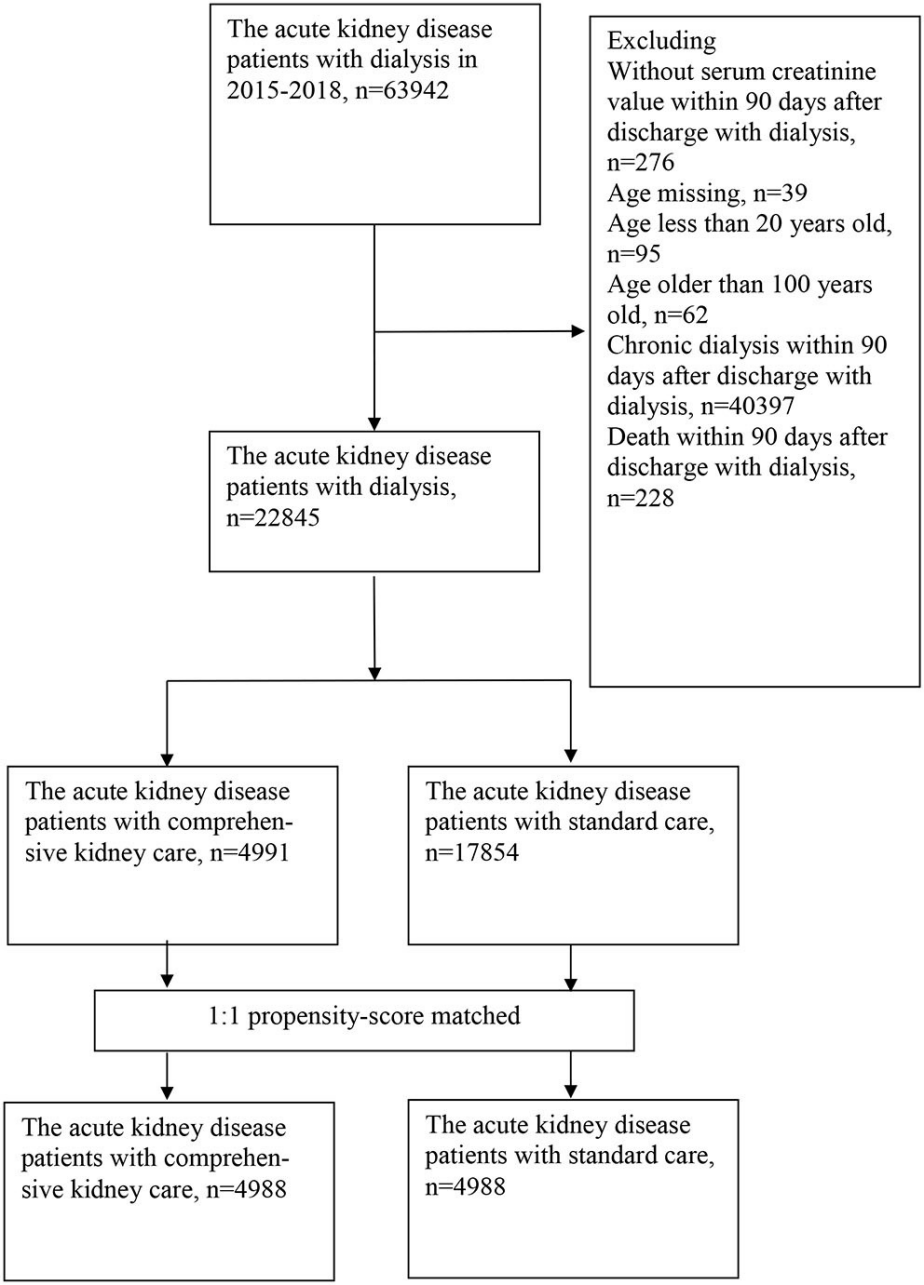
Flow chart of patient selection.

From January 2015 through December 2018, we identified 22,845 AKI patients who had recovered from acute dialysis and survived (Figure 1). Among them, 4,991 patients were enrolled into the comprehensive kidney care group, and the other 17,854 were enrolled in the standard care group. A large proportion of patients were excluded due to starting dialysis within 90 days after discharge (*n* = 40,397). This is a feature of the national health insurance in Taiwan. All patients receiving dialysis must apply for “Catastrophic illness” to receive full reimbursement of dialysis fees. The first application for the Catastrophic illness will give patients 3 months of full reimbursement, regardless of whether dialysis was induced by chronic deterioration of CKD or AKI. The comparison of the characteristics of included cases and excluded cases are available in Supplementary Table S1. The initial analysis of the cohort showed that those patients in the standard care group were older and had higher baseline serum creatinine levels. Because residual renal function is an important predictor of renal prognosis, we subsequently performed propensity match 1:1 analysis including estimated glomerular filtration rate (eGFR) based on CKD-EPI to eliminate confounding factors (Figure 1).

After the propensity match, each cohort had 4,988 patients. The baseline characteristics, including age, sex, serum creatinine, eGFR, diabetes, and chronic kidney disease were all balanced across the two groups (Table 1). Their mean age was 73 years, with 4,045 female participants (40.5%). Their mean eGFR based on CKD-EPI was 27.5 ml/min/1.73 m^2^, and 6,668 patients (66.7%) had type 2 diabetes. The mean follow-up was 1.4 years.

**Table 1:** Demographic characteristics of dialysis-requiring acute kidney injury survivors receiving comprehensive kidney care or standard care.

	**Before propensity score matched**				**After propensity score inverse probability weighting matched**			
	**Comprehensive kidney care**	**Standard care**	**SMD**	***P*-value**	**Comprehensive kidney care**	**Standard care**	**SMD**	***P*-value**
*N*	4,991	17,854			4,988	4,988		
**Age groups, years old**								
Age 20–39	75 (1.5)	332 (1.9)	0.02776	0.09	75 (1.5)	72 (1.4)	0.00499	0.8
Age 40–64	1,122 (22.5)	3,844 (21.5)	0.02294	0.15	1,121 (22.5)	1,137 (22.8)	0.00767	0.7
Age 65–74	1,241 (24.9)	3,941 (22.1)	0.0659	<0.001	1,239 (24.8)	1,242 (24.9)	0.00139	0.94
Age 75+	2,553 (51.2)	9,737 (54.5)	0.06784	<0.001	2,553 (51.2)	2,537 (50.9)	0.00642	0.75
Mean (SD)	73 (12.5)	73.6 (13.5)	0.04612	0.001	73 (12.5)	72.9 (13.2)	0.00778	0.73
**Gender**			·	<0.001				0.95
Male	2,970 (59.5)	9,980 (55.9)	0.07311		2,967 (59.5)	2,964 (59.4)	0.00122	
Female	2,021 (40.5)	7,874 (44.1)	0.07311		2,021 (40.5)	2,024 (40.6)	0.00122	
**Comorbidity**								
HTN	4,137 (82.9)	14,820 (83)	0.00312	0.85	4,133 (82.8)	4,151 (83)	0.00386	0.85
Diabetes	3,711 (74.4)	11,467 (64.2)	0.22088	<0.001	3,342 (67)	3,325 (66.5)	0.01071	0.59
Dyslipidemia	1,914 (38.3)	5,462 (30.6)	0.16375	<0.001	1,632 (32.7)	1,616 (32.3)	0.00834	0.68
CVA	1,104 (22.1)	3,846 (21.5)	0.014	0.38	1,095 (22)	1,084 (21.7)	0.0068	0.73
CHF	1,564 (31.3)	5,475 (30.7)	0.01451	0.36	1,522 (30.5)	1,539 (30.8)	0.00554	0.78
CVD	1,737 (34.8)	5,986 (33.5)	0.02689	0.09	1,679 (33.7)	1,689 (33.8)	0.00226	0.91
HBV	195 (3.9)	564 (3.2)	0.04053	0.009	194 (3.9)	189 (3.8)	0.00522	0.79
HCV	209 (4.2)	524 (2.9)	0.06763	<0.001	207 (4.1)	214 (4.3)	0.00698	0.73
COPD	852 (17.1)	2,766 (15.5)	0.04276	0.007	851 (17.1)	875 (17.5)	0.01272	0.53
AKI	2,713 (54.4)	8,743 (49)	0.10799	<0.001	2,534 (50.8)	2,510 (50.2)	0.01198	0.55
Cancer	1,121 (22.5)	3,433 (19.2)	0.07964	<0.001	1,119 (22.4)	1,056 (21.2)	0.03059	0.13
**CCI score**								
Mean (SD)	5.8 (2.4)	5.5 (2.4)	0.12500	<0.001	5.8 (2.4)	5.8 (2.4)	<0.0001	0.52
**Creatinine (mg/dL)**								
Mean (SD)	3.7 (3.1)	5.1 (3.5)	0.42347	<0.001	3.7 (3.1)	3.7 (2.9)	<0.0001	0.78
**CKD-EPI (eGFR, min/ml/1.73 m** ^**2**^ **)**								
Mean (SD)	27.5 (24.4)	18.7 (21.7)	0.38113	<0.001	27.5 (24.4)	27.5 (25.1)	<0.0001	0.95
**CKD stage**								
3A	434 (8.7)	629 (3.5)	0.21725	<0.001	432 (8.7)	412 (8.3)	0.01441	0.47
3B	712 (14.3)	1,098 (6.1)	0.27051	<0.001	711 (14.3)	716 (14.4)	0.00286	0.89
4	1,179 (23.6)	3,320 (18.6)	0.12343	<0.001	1,179 (23.6)	0.6)	<0.0001	0.99
5	2,118 (42.4)	11,652 (65.3)	0.47038	<0.001	2,118 (42.5)	2,122 (42.5)	0.00162	0.94
Received care in past 1 year	2,377 (47.6)				2,375 (47.6)			
Received care in 90 days after acute kidney disease with dialysis	314 (6.3)				314 (6.3)			
Propensity score	0.3 (0.1)	0.2 (0.1)	1.0000	<0.001	0.3 (0.1)	0.3 (0.1)	<0.001	0.93

*SMD, Standardized mean difference; SD, Standard Deviation; HTN, Hypertension; CVA, cerebrovascular accident; CHF, congestive heart failure; CVD, cardiovascular disease; HBV, hepatitis B virus; HCV, hepatitis C virus; COPD, chronic obstructive pulmonary disease; AKI, Acute Kidney Injury*.

### Efficacy Outcomes

**Figure 2:**
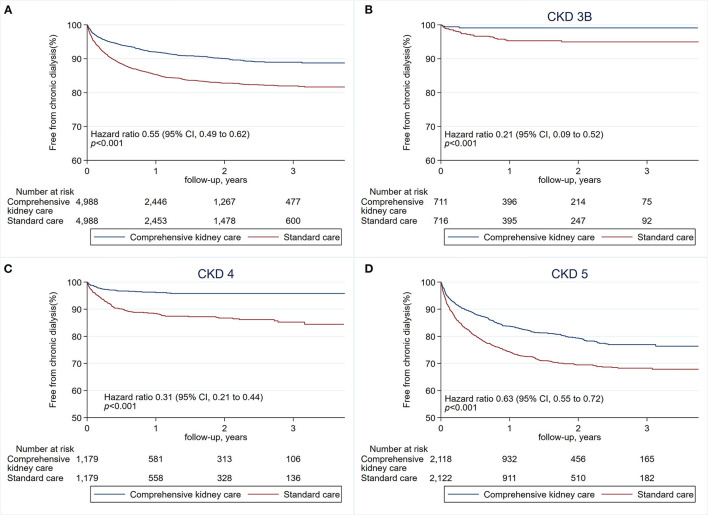
Risks of chronic dialysis or end-stage renal disease (ESRD). The first primary outcome is the risk of chronic dialysis or ESRD **(A)**, estimated with the Kaplan–Meier method. Hazard ratios, confidence intervals, and *P*-values were estimated with the Cox proportional-hazards regression model. Risks of chronic dialysis and ESRD when stratified according to chronic kidney disease (CKD) stage 3B **(B)**, CKD stage 4 **(C)**, and CKD stage 5 **(D)**.

The first primary outcome analyzed was the risk of chronic dialysis or end-stage renal disease (ESRD) estimated with the Kaplan–Meier method. Hazard ratios, confidence intervals, and *P*-values were estimated with the Cox proportional-hazard regression model (Table 2). The risk of chronic dialysis increased with CKD severity. The event rates favored the comprehensive kidney care group. Specifically, in comparing the two groups, the hazard ratio was 0.55 (95% confidence interval [CI] 0.49–0.62, *p* < 0.001). The 45% drop in risks of chronic dialysis risks via the comprehensive kidney care group was seen early in the follow-up, with persistent benefits for patients with CKD stages 4 and 5. The beneficial effects were generally consistent when stratified according to chronic kidney disease (CKD) stages 3B, 4, and 5. The three stages' respective hazard ratio was 0.21 (95% CI, 0.09–0.52; *p* = 0.001), 0.31 (95% CI, 0.21–0.44; *p* < 0.001), 0.63 (95% CI, 0.55–0.72; *p* < 0.001), indicating those patients with CKD stage 3B and stage 4 were benefited most (Table 3, Figure 2).

**Table 2:** Outcomes of dialysis-requiring acute kidney injury survivors receiving comprehensive kidney care or standard care.

	**Comprehensive kidney care**		**Standard care**		**Comprehensive kidney care vs. Standard care**	
	**Event case number**	**Incidence rate**	**Event case number**	**Incidence rate**	**Crude HR (95% confidence intervals)**	***P*** **-value**
Chronic dialysis	379	60.46	3,384	149.47	0.39 (0.35–0.43)	<0.001
All-cause mortality	1,040	154.69	4,483	163.51	0.89 (0.83–0.95)	<0.001
**After propensity score matced**
Chronic dialysis	379	60.52	684	104.41	0.58 (0.54–0.62)	<0.001
All-cause mortality	1,040	154.83	1,381	186.02	0.83 (0.74–0.92)	<0.001

**Table 3:** Risks of dialysis or all-cause mortality.

**Risk of chronic dialysis in patients with comprehensive kidney care**
**CKD stage**	**aHR**	**95 % CI**	***p***
All	0.55	0.49–0.62	<0.001
CKD 3B	0.21	0.09–0.52	0.001
CKD 4	0.31	0.21–0.44	<0.001
CKD 5	0.63	0.55–0.72	<0.001
**Risk of all-cause mortality in patients with comprehensive kidney care**
CKD stage	aHR	CI	
All	0.79	0.73–0.86	<0.001
CKD 3B	0.71	0.57–0.88	0.002
CKD 4	0.69	0.60–0.81	<0.001
CKD 5	0.82	0.73–0.93	0.003

*CKD, chronic kidney disease; aHR, adjusted hazard ratios; CI, confidence interval*.

**Figure 3:**
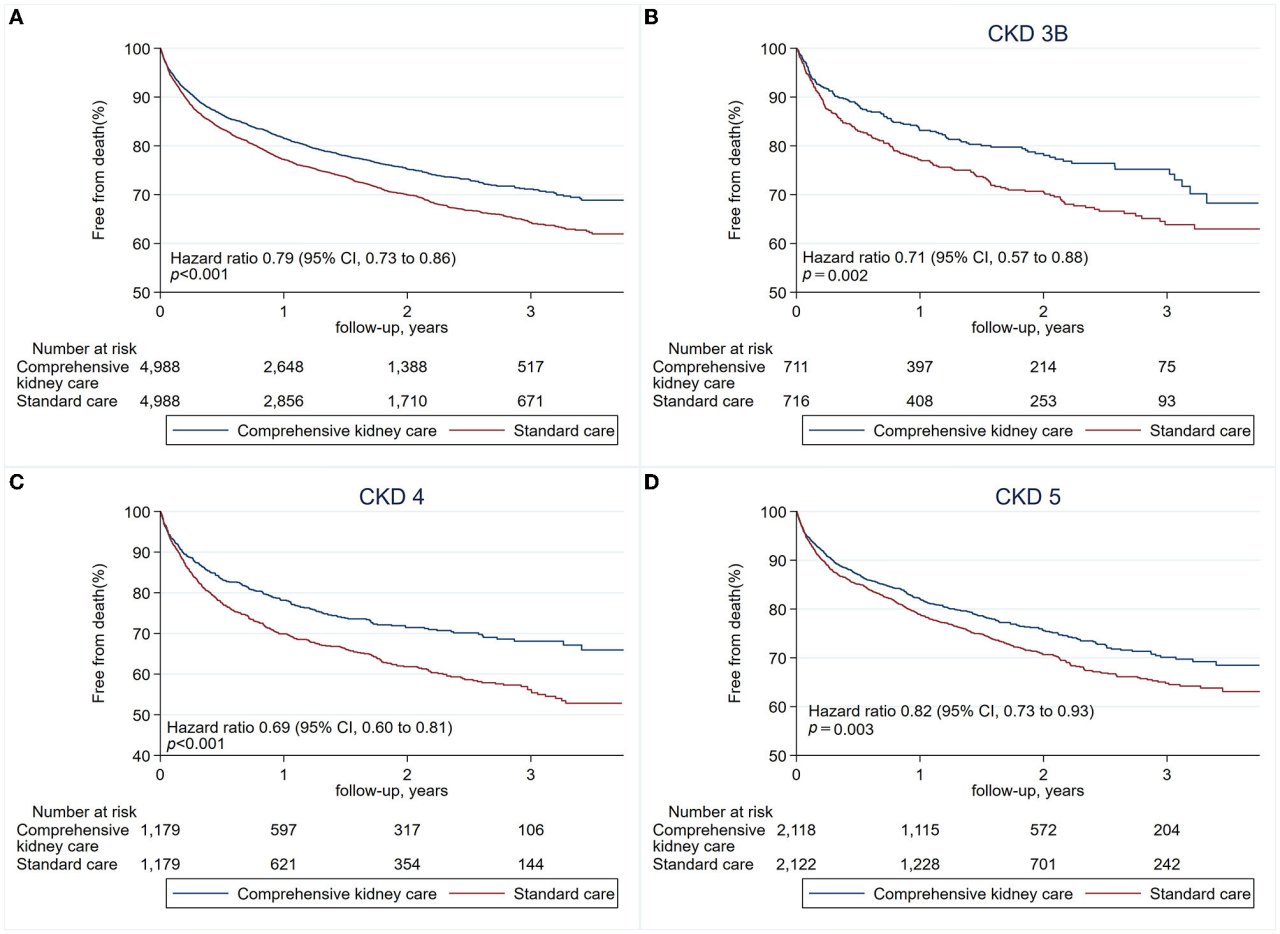
Risks of all-cause mortality. The second primary outcome is the risk of all-cause mortality **(A)**, estimated with the Kaplan–Meier method. Hazard ratios, confidence intervals, and *P*-values were estimated with the Cox proportional-hazards regression models. Risk of mortality when stratified according to chronic kidney disease (CKD) stage 3B **(B)**, CKD stage 4 **(C)**, and CKD stage 5 **(D)**.

The second primary outcome analyzed was all-cause mortality, as estimated with the Kaplan–Meier method. Hazard ratios, confidence intervals, and *P*-values were estimated with the Cox proportional-hazard regression model (Table 2). The finding on mortality rate favored the comprehensive kidney care. In comparing the two groups, the hazard ratio was 0.79 (95% confidence interval [CI], 0.73–0.86; *p* < 0.001). The 21% drop in the risk of mortality was consistent when stratified according to stages of chronic kidney disease (CKD) (stages 3B, 4, and 5). The three stages' respective hazard ratio was 0.71 (95% CI, 0.57–0.88; *p* = 0.002), 0.69 (95% CI, 0.60–0.81; *p* < 0.001), 0.82 (95% CI, 0.73–0.93; *p* = 0.003). Patients with CKD stage 3B and 4 had the most drop in mortality, whereas those with stage 5 were least benefited (Table 3, Figure 3).

In addition, we performed a subgroup analysis based on the etiology of the D-AKI during admission. The comprehensive kidney care group had a lower risk of chronic dialysis and all-cause mortality when compared to standard care patients if the etiology of the D-AKI was infectious of cardiovascular (Supplementary Table S2).

## Discussion

Nephrology follow-up is the lynchpin of continued quality care of AKI as proposed by the Acute disease quality initiative (ADQI) (13). Our study provided additional evidence that early intervention via comprehensive kidney care, centered on the nephrologist with coordinated multidisciplinary kidney care team improves renal outcomes and mortality of survivors of dialysis-requiring acute kidney injury.

The 45% drop in risks of chronic dialysis could delay detrimental effects of hemodialysis on health-related quality of life (30), and reduce the heavy work burden of caregivers (31, 32). Lower incidence of chronic dialysis also eases the financial burden that is perpetually straining the health care system (17, 18, 20). The beneficial effect was especially pronounced in those survivors of dialysis-requiring acute kidney injury diagnosed with CKD stage 3B stage 4 during the initial follow-up period, with a steep reduction in risks of chronic dialysis of 79 and 69% respectively.

Compared to the study of Harel et al., who had demonstrated that early nephrology follow-up, within 90 days of discharge, resulted in 24% reduction in mortality during a mean follow-up of 2 years. We found similarly a 21% reduction in mortality, and also a lower risk of chronic dialysis. Although such findings of lower mortality are comparable in these two studies, we emphasized the importance of a kidney care team, leading to the additional benefit of a lower risk of chronic dialysis. In addition, CKD stage 3B and 4 also had the best gains in all-cause mortality, at 29 and 31% respectively.

Our study shows that early intervention has a great impact on the risks of chronic dialysis and survival, as depicted by the early diversion of the Kaplan–Meier curves. In one study on 16,968 patients with AKI stage 2 or 3, patients in early recovery had marked improvement on 1-year survival compared with those who never recovered their kidney function (90.2 vs. 39.2%) or those with relapsed AKI (41.9% 1-year survival) (33). In another recent study of 47,903 patients, those with a protracted course of recovery had a higher risk of losing their kidney function shortly after recovery (34).

The importance of early intervention cannot be overemphasized, as shown in the study by Silver et al. For AKI survivors who were previously hospitalized, their readmission rate was 18% during the next 30 days. Of them, 10% visited the emergency department and 5% died (35).

Previous studies reported that patients under comprehensive kidney care were more likely to receive statin medications, more likely to be under Renin-Angiotensin-Aldosterone System Blockade if hypertensive, and less likely to receive NSAIDs (15, 36–38). Follow-up by nephrologists also increased the use of diuretics and anti-diabetic medications. Moreover, these patients were more likely to be examined with sonography (15). The strength of our study is that it is population based, with laboratory data, like serum creatinine level. Our study reflects real-world circumstances, with recently obtained laboratory data, making the propensity match more accurate. In addition, much has changed since the introduction of the KDIGO guidelines on acute kidney injury. Compared with previous studies, our patient population is a more recent cohort, with contemporary practices more compatible with the 2012 KDIGO AKI guidelines.

The FUSION trial also emphasized on early nephrologist follow-up, but could not overcome feasibility barriers (16). Among the 34 patients assigned to the intervention group, 10 of them were not followed up by the nephrologist with success, making results less conclusive. We are very fortunate that the foundations for comprehensive follow-up via pre-ESRD program were already established by 2006. Widespread implementation was made possible by herculean efforts by Taiwan's National Health Insurance and the Taiwan Society of Nephrology.

Many prediction models and risk models have been proposed for post-AKI follow-up (39, 40), our study showed that for survivors of dialysis-requiring acute kidney injury, post-discharge CKD stage may profound impact on renal survival and all-cause mortality. Risk stratification using this method is practical, requiring no additional calculation. It is an easy approach to educate general practitioners and the general public on the substantial risks of chronic dialysis and death in survivors of D-AKI.

An omnipresent challenge with retrospective studies is the involvement of potential confounders. Through laboratory data and propensity matching, we had minimized the influence of these confounders. In addition, multiple comorbidities may hinder patients from receiving comprehensive kidney that is time consuming and may require more frequent visits. However, the comorbidities are matched in our cohorts, minimizing such biases. In addition, the standard care group had a higher proportion of CKD stage 4 and stage 5 patients prior to propensity matching. Although there may be concerns of selection bias, we used the nationwide registered data from the NHI. The NHI is the sole insurance carrier of the covered healthcare in Taiwan, with 99% coverage of the whole population. Therefore, it should have a representative sample and the selection bias if present may be mild. We also stratified patients according to CKD stage for the analysis, and noted CKD stage 3B and 4 had the greatest gains in risks of chronic dialysis and all-cause mortality.

Another limitation is that our study only evaluated patients with D-AKI, but not patients with AKI stage 2 or stage 3. This is because the National Health Insurance Research Database in Taiwan currently does not include urine output records. We believe post-discharge AKI care represents an opportunity to improve outcomes for all AKI patients. Further studies are needed to address the impact of comprehensive kidney care on AKI patients with mild stages of disease.

Finally, prospective randomized control trials could further determine the critical components that improve outcomes of chronic kidney disease and mortality.

## Conclusions

Among survivors of dialysis-requiring acute kidney injury, those patients who received early comprehensive kidney care via nephrologist together with coordinated multidisciplinary kidney team had a significantly lower risk for chronic dialysis or all-cause mortality.

## Data Availability Statement

The datasets presented in this article are not readily available because the data underlying this article are stored in the National Health Insurance Research Database (NHIRD) of Taiwan. It can only be accessed via direct approval via the NHIRD. Requests to access the datasets should be directed to sgazn.tw@gmail.com.

## Ethics Statement

The studies involving human participants were reviewed and approved by Research Ethics Committee of the National Health Research Institutes in Taiwan. Written informed consent for participation was not required for this study in accordance with the national legislation and the institutional requirements.

## Author Contributions

C-YW, J-SL, C-CH, and M-JW: conception/design. C-YW, J-SL, C-TH, C-CH, and M-JW: provision of study materials. C-CH and J-SL: collection and/or assembly of data. All authors: data analysis and interpretation, manuscript writing, and final approval of manuscript. All authors have contributed significantly and are in agreement with the content of the manuscript.

## Conflict of Interest

The authors declare that the research was conducted in the absence of any commercial or financial relationships that could be construed as a potential conflict of interest.

## Publisher's Note

All claims expressed in this article are solely those of the authors and do not necessarily represent those of their affiliated organizations, or those of the publisher, the editors and the reviewers. Any product that may be evaluated in this article, or claim that may be made by its manufacturer, is not guaranteed or endorsed by the publisher.
