# Supplementary material for: Early Comprehensive Kidney Care in Dialysis-Requiring Acute Kidney Injury Survivors: A Populational Study
Source: Front Med (Lausanne). 2022 Apr 22;9:847462. doi: 10.3389/fmed.2022.847462 (PMC9072865; doi:10.3389/fmed.2022.847462)
Supplement: Supplementary file 1 [file Table_1.DOCX]

Table S1. Demographic characteristics of dialysis-requiring acute kidney injury in included and excluded cases

|  | included cases | excluded cases | p-value |
| --- | --- | --- | --- |
|  | 22845 | 40625 |  |
| Age groups, years old |  |  |  |
| age 20-39 | 407 (1.8) | 1467 (3.6) | <0.001 |
| age 40-64 | 4966 (21.7) | 14504 (35.7) | <0.001 |
| age 65-74 | 5182 (22.7) | 10669 (26.3) | <0.001 |
| age 75+ | 12290 (53.8) | 13985 (34.4) | <0.001 |
| Mean (SD) | 73.5 (13.3) | 67.5 (14) | <0.001 |
| Gender |  |  |  |
| male | 12950 (56.7) | 22727 (55.9) | 0.07 |
| female | 9895 (43.3) | 17898 (44.1) | 0.07 |
| Comorbidity |  |  |  |
| HTN | 18957 (83) | 37417 (92.1) | <0.001 |
| Diabetic | 15178 (66.4) | 26301 (64.7) | <0.001 |
| Hyperlipidemia | 7376 (32.3) | 15083 (37.1) | <0.001 |
| Stroke | 4950 (21.7) | 5649 (13.9) | <0.001 |
| CHF | 7039 (30.8) | 11246 (27.7) | <0.001 |
| CVD | 7723 (33.8) | 11314 (27.8) | <0.001 |
| HBV | 759 (3.3) | 1148 (2.8) | 0.001 |
| HCV | 733 (3.2) | 1026 (2.5) | <0.001 |
| COPD | 3618 (15.8) | 3424 (8.4) | <0.001 |
| AKI | 639 (2.8) | 1735 (4.3) | <0.001 |
| Cancer | 4554 (19.9) | 4415 (10.9) | <0.001 |
| Creatinine (mg/dL) | 4.8 (3.5) | 8.1 (3.8) | <0.001 |
| Mean (SD) |  |  |  |
| CKD-EPI eGFR, min/ml/ 1.73 m^2^ |  |  |  |
| Mean (SD) | 20.6 (22.6) | 8.4 (10.5) | <0.001 |
| CKD stage |  |  |  |
| 3a | 1063 (4.7) | 259 (0.6) | 0.25221 |
| 3b | 1810 (7.9) | 446 (1.1) | 0.33341 |
| 4 | 4499 (19.7) | 2471 (6.1) | 0.41487 |
| 5 | 13770 (60.3) | 37010 (91.1) | 0.77003 |
| AKI dialysis cause, n(%) |  |  |  |
| Infectious/septic | 7867 (41.6) | 11718 (42.1) | 0.00988 |
| Cardiovascular | 13449 (71.2) | 18959 (68.1) | 0.06583 |
| Post-surgical | 331 (1.8) | 718 (2.6) | 0.05698 |
| Organophosphate poisoning or Heavy metal exposure | 47 (0.2) | 40 (0.1) | 0.02371 |

SD: Standard Deviation

HTN: Hypertension

CVA: cerebrovascular accident

CHF: congestive heart failure

CVD: cardiovascular disease

HBV: hepatitis B virus

HCV: hepatitis C virus

COPD: chronic obstructive pulmonary disease

AKI: Acute Kidney Injury

| Table S2. The risks of chronic dialysis and all-cause mortality by AKI etiology | | | | | | | | |
| --- | --- | --- | --- | --- | --- | --- | --- | --- |
|  | Comprehensive  Kidney care | | Standard care | | Comprehensive kidney care vs. Standard care |  | Comprehensive kidney care vs. Standard care |  |
|  | event | Incidence rate (per 1000 person-years) | Event | Incidence rate (per 1000 person-years) | Crude hazard ration (95% confidence intervals) | P value | Adjusted hazard ration (95% confidence intervals) | P value |
| Chronic dialysis |  | | | | | | | |
| Infectious/septic | 149 | 80.47 | 1096 | 221.74 | 0.37 (0.32-0.44) | <.001 | 0.51 (0.43-0.60) | < 0.001 |
| Cardiovascular | 230 | 49.39 | 2354 | 128.68 | 0.39 (0.34-0.44) | <.001 | 0.52 (0.46-0.60) | < 0.001 |
| Post-surgical | 15 | 259.07 | 63 | 333.07 | 0.76 (0.43-1.33) | 0.33 | 0.97 (0.53-1.78) | 0.93 |
| Organophosphate poisoning or Heavy metal exposure | <6 | 93.63 | <6 | 168.86 | 0.66 (0.08-5.69) | 0.70 | - | - |
| All-cause mortality |  | | | | | | | |
| Infectious/septic | 432 | 218.42 | 1423 | 240.41 | 0.90 (0.81-1.00) | 0.06 | 0.88 (0.79-0.99) | 0.027 |
| Cardiovascular | 650 | 129.93 | 3248 | 146.14 | 0.87 (0.80-0.95) | 0.001 | 0.83 (0.76-0.91) | < 0.001 |
| Post-surgical | 22 | 302.85 | 73 | 288.96 | 1.03 (0.64-1.67) | 0.89 | 1.26 (0.75-2.11) | 0.39 |
| Organophosphate poisoning or Heavy metal exposure | <6 | 82.41 | <6 | 86.34 | 0.69 (0.06-7.64) | 0.76 | - | - |
